# Central retinal vein occlusion associated with *Bartonella henselae* infection

**DOI:** 10.1186/s12348-023-00334-5

**Published:** 2023-03-28

**Authors:** Sunil Bellur, Amir Ali, Nam V. Nguyen, Joshua K. Fernandes, Shilpa Kodati

**Affiliations:** 1 National Eye Institute, National Institutes of Health, 10 Center Dr, 10/10N248, Bethesda, Maryland 20892 USA; 2 University of Texas Medical Branch, Galveston, Texas USA; 3 College of Medicine, University of Nebraska Medical Center, Omaha, Nebraska USA; 4 DC Retina, Silver Spring, Maryland USA

**Keywords:** Central retinal vein occlusion, Macular edema, Vasculitis, Cat scratch disease, Ocular bartonellosis, *Bartonella henselae*

## Abstract

**Purpose:**

To report the clinical features and treatment course of a case of central retinal vein occlusion (CRVO) as the initial sign of ocular *Bartonella henselae* (*B. henselae*) infection.

**Observation:**

A 36-year-old male was evaluated for unilateral vision loss. He denied prodromal symptoms but reported prior exposure to fleas. Best corrected visual acuity (BCVA) was 20/400 in the left eye. Clinical examination revealed a CRVO with atypical features including significant peripapillary exudates and peripheral vascular sheathing. Laboratory testing revealed elevated *B. henselae* IgG titers (1:512) with no abnormalities on hypercoagulability testing. The patient was treated with doxycycline and aflibercept with an excellent clinical response and improvement in BCVA to 20/25 in the left eye two months later.

**Conclusion:**

CRVO is a rare but sight-threatening complication of ocular bartonellosis and can be the presenting sign of infection, even in the absence of cat exposure or prodromal symptoms.

## Introduction

*Bartonella henselae* (*B. henselae*) is a gram-negative, intracellular bacteria that is the causative agent of cat-scratch disease (CSD). Transmission of *B. henselae* typically occurs through cat scratches, bites, or wound contamination with over 90% of CSD cases reporting a history of exposure to cats; however, direct human exposure to the cat flea (*Ctenocephalides felis*) or its feces has also been implicated in disease pathogenesis [1–3]. The clinical presentation of CSD is variable and ranges from mild self-limited disease to atypical cases that include ocular findings [4].

Ocular bartonellosis is uncommon and occurs in 5–10% of patients [5]. While the most common ocular manifestation is Parinaud’s oculoglandular syndrome, other reported findings include vascular and inflammatory changes such as retinal vascular occlusions, neuroretinitis, vitritis, retinitis, retinal vasculitis, serous retinal detachments, and choroiditis [6, 7]. The prevalence of retinal vascular occlusions in ocular bartonellosis is variable, with one review article reporting a rate of 4–23% [8]. The majority of vascular occlusions involve a branch artery or vein, with central retinal vein occlusion (CRVO) due to *B. henselae* infection being exceedingly rare (two reported adult cases) and associated with broader, severe ischemic ocular disease [9, 10]. We report a unique case of a 36-year-old patient with no exposure to cats or prodromal illness who presented with a CRVO as the only clinical sign, with subsequent serologic testing revealing *B. henselae* infection. The patient had excellent visual recovery with oral doxycycline and anti-vascular endothelial growth factor (anti-VEGF) therapy.

## Case report

**Fig. 1:**
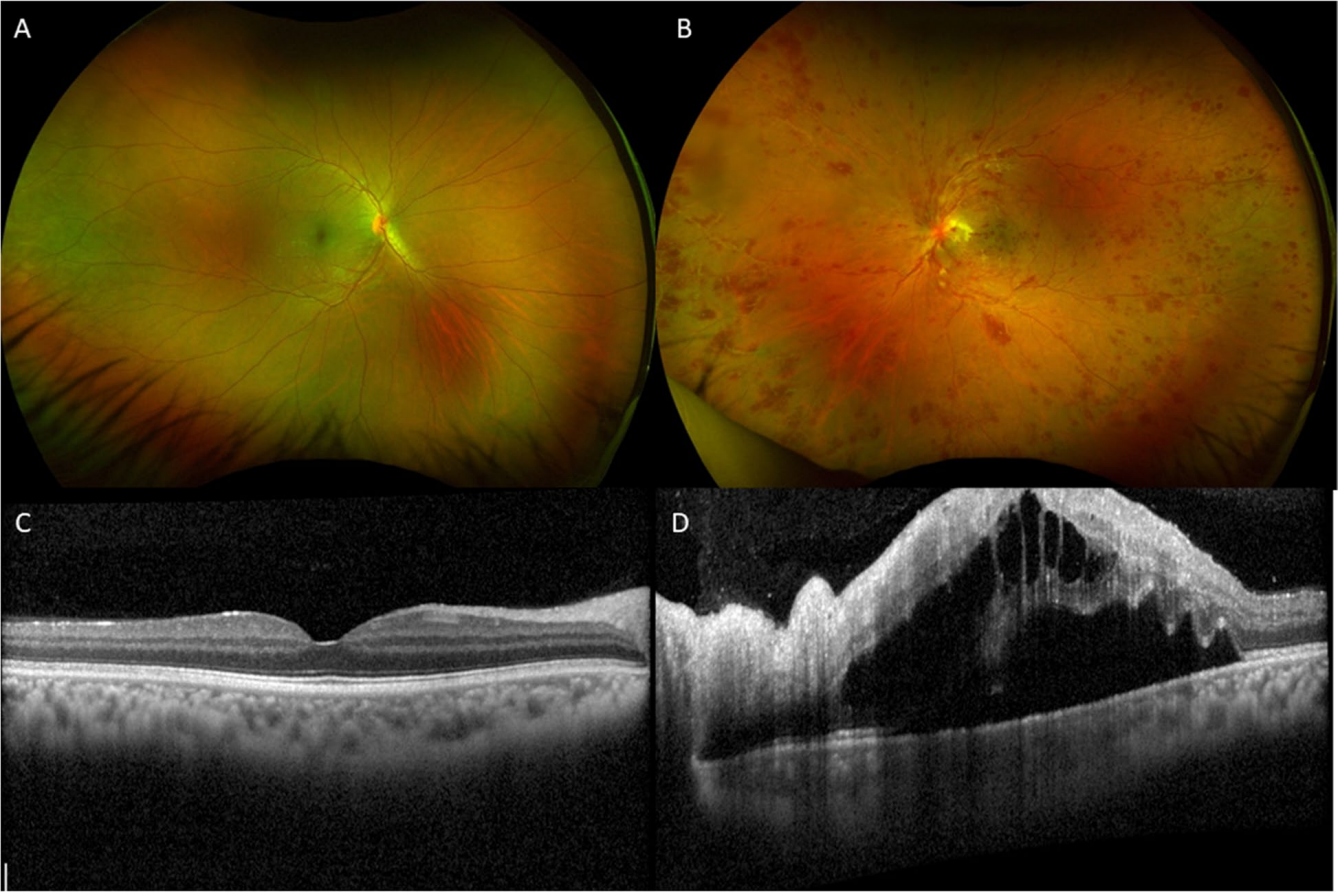
Wide-field color fundus photos demonstrating normal findings in the right eye (**A**), and optic disc edema, macular edema with peripapillary exudates, venous engorgement with vascular sheathing in nasal periphery, and diffuse hemorrhages consistent with a central retinal vein occlusion in the left eye (**B**). Optical coherence tomography shows no abnormalities in the right eye (**C**), and prominent subretinal and cystic intraretinal fluid with inner retinal thickening nasally in the left eye (**D**)

**Fig. 2:**
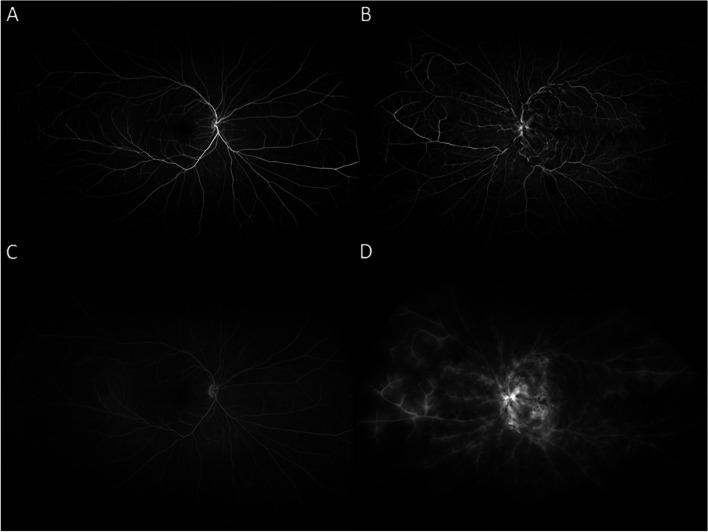
Wide-field fluorescein angiography shows no retinal vascular leakage in the right eye (**A**, **C**). In the left eye there is peripheral ischemia seen in early and late frames with disc leakage, macular leakage, and diffuse, large vessel, vascular leakage predominantly affecting the venules (**B**, **D**)

A 36-year-old Ethiopian male presented with a 10-day history of acute, painless vision loss in the left eye (OS). A review of systems was negative. His past medical history was notable for hiatal hernia related gastric ulcers; there was no history of vascular risk factors apart from prior tobacco use. The patient denied exposure to cats but lived on farmland where fleas were reportedly endemic. On evaluation, his best corrected visual acuities (BCVA) were 20/20 in his right eye (OD) and 20/400 in the left eye (OS). A 1 + relative afferent pupillary defect OS was present. Anterior segment exam was notable for trace pigmented cell in the anterior chamber OS. Fundus examination of the left eye revealed trace pigmented anterior vitreous cell with no haze, optic disc edema, macular edema with exudates in the nasal macula, scattered cotton wool spots, mildly increased venular engorgement with peripheral vascular sheathing, and diffuse intraretinal hemorrhages in the macula and all four quadrants of the periphery (Fig. 1B). Optical coherence tomography (OCT) of the macula showed subretinal fluid with cystoid macular edema (CME) OS (Fig. 1D). Fluorescein angiography revealed leakage of the disc and macula with peripheral capillary ischemia and diffuse vessel leakage consistent with a predominant phlebitis OS (Fig. 2B, D). The findings were consistent with an ischemic central retinal vein occlusion OS. Clinical exam and multimodal imaging in the right eye were unremarkable (Figs. 1 A, C and 2 A, C).

Given the patient’s young age and lack of vascular risk factors, a laboratory workup was performed to identify potential underlying precipitating factors such as coagulation disorders, infection, or inflammatory diseases. The blood pressure was 132/84, hemoglobin A1c was 5.0%, and no dyslipidemia was noted on lipid panel testing. An extensive hypercoagulable workup was unrevealing including normal homocysteine levels, protein C/S levels, antithrombin III activity, anti-cardiolipin levels, and absence of anti-proteinase-3 and anti-myeloperoxidase antibodies. No prothrombin 20210 or factor V leiden mutation was detected. Infectious workup revealed positive *Bartonella henselae* IgG with elevated titers of 1:512 on immunofluorescence assay testing (Mayo Clinic Laboratories, Rochester, MN). IgM antibodies were negative. Syphilis total antibody testing, quantiFERON-TB Gold, and HIV 1/2 testing were all negative. *Toxoplasma gondii* IgG antibodies were mildly positive (84 International Units/mL) while no IgM antibodies were detected.

The patient was diagnosed with a CRVO in the setting of *Bartonella henselae* infection based on high *B. henselae* IgG titers, atypical presentation including the observed inflammatory findings, and the absence of co-existent risk factors. The patient was started on doxycycline 100 mg PO BID and treated with monthly aflibercept injections for the CME. Initiation of oral steroids was deferred given the patient’s concurrent gastric ulcers.

**Fig. 3:**
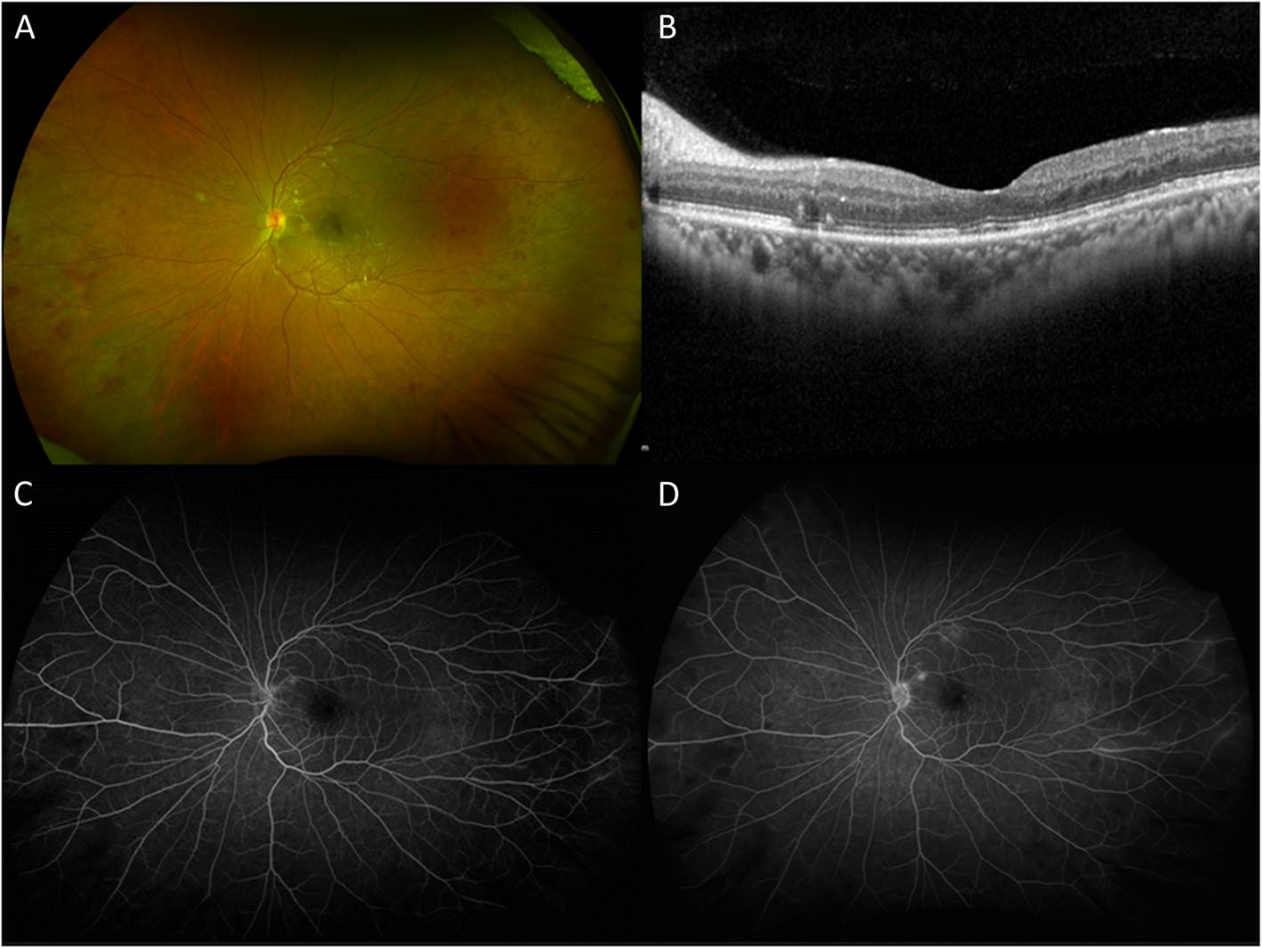
Repeat evaluation 2-months after presentation and treatment initiation shows resolution of optic disc edema and improvement of retinal hemorrhages (**A**). Optical coherence tomography shows resolution of subretinal and intraretinal fluid with residual focal ellipsoid zone irregularities (**B**). There is near resolution of leakage on fluorescein angiogram with persistent but improved peripheral ischemia during mid-phase (**C**) and late frames (**D**)

Repeat examination two months after completing a 4-week course of doxycycline and receiving two aflibercept injections showed improvement in the left eye with a BCVA of 20/25 with resolution of pigmented anterior chamber cell and stable trace pigmented vitreous cell. Multimodal imaging of the left eye revealed improvement of the retinal hemorrhages with resolution of optic disc edema and vascular sheathing (Fig. 3A). Repeat OCT of the macula showed resolution of subretinal fluid and cystoid macular edema with residual focal ellipsoid zone irregularities (Fig. 3B). On fluorescein angiography there was resolution of disc and macular leakage along with resolved vascular leakage; there was improved but persistent peripheral ischemia (Fig. 3C, D). Repeat *B. henselae* titers revealed lower IgG titers (1:256) and negative IgM titers.

## Discussion

We report a rare case of CRVO associated with *B. henselae* infection confirmed by serological testing. Our patient lacked the preceding contact to cats but did have exposure to arthropod vectors such as fleas. The CRVO was suspected to be inflammatory in etiology given the patient’s young age, absence of vascular and hypercoagulable risk factors, and clinical features such as vascular sheathing, severe phlebitis on fluorescein angiogram, and prominent peripapillary exudates. There was an excellent clinical response to anti-VEGF agents and oral doxycycline.

The clinical presentation of *B. henselae* infection can involve multiple organ systems. Systemic findings seen in typical CSD can include transient papules or pustules at the inoculation site followed by regional lymphadenopathy with or without fever [11]. Other findings are broad and can range from constitutional symptoms such as malaise, nausea, vomiting and anorexia to organic specific findings such as hepatomegaly, splenomegaly, endocarditis, hemolytic anemia, thrombocytopenic purpura, glomerulonephritis, and osteomyelitis [5, 11]. Immunocompromised patients are at risk of bacillary angiomatosis, altered mental status and dementia [5].

Ocular manifestations of *B. henselae* infection are varied and can affect numerous ocular structures. While Parinaud’s oculoglandular syndrome with fever, granulomatous conjunctivitis, and regional lymphadenopathy is the most common ocular finding, posterior segment manifestations including neuroretinitis, retinochoroiditis, retinitis, macular hole, serous retinal detachments, vitritis, vasculitis, papillitis, retinal bacillary angiomatosis, subretinal vascular masses, uveitis, and retinal vascular occlusions have been reported in the literature [2, 6, 7]. Bilateral ocular involvement has been described in 17–24% of patients; 58% of ocular bartonellosis patients reported fever and 77% had malaise and/or weakness in one retrospective study by Habot-Wilner and colleagues [5, 6].

Reported literature cites branch retinal vascular occlusions as the most common variant of vascular occlusion in ocular bartonellosis and may be the presenting sign of disease [6, 8]. In a 20-year retrospective study, 8/107 (7%) of eyes had a retinal vascular occlusion; four eyes had a branched retinal artery occlusion (BRAO), three had branched retinal vein occlusion (BRVO), and one patient had a combined BRAO and BRVO. In one case, the BRVO was the only manifestation of CSD [6]. A case series from Greece of 14 eyes of eight patients with ocular bartonellosis noted one 36-year-old patient with a BRVO and periphlebitis on fluorescein angiography and an IgG titer of 1:32. Treatment with rifampin and azithromycin lead to an improvement in visual acuity and macular edema [11]. Eiger-Moscovich *et* al. showed six, young, otherwise healthy patients with a BRAO due to *B. henselae* infection. Four patients had an exposure to cats, while one patient had a history of flea bites and another with no exposure identified; all patients had a single highly elevated IgG or IgM titer for *B. henselae* [12]. A study of 35 eyes with ocular bartonellosis with posterior segment findings reported a retinal vascular occlusion in 14% of eyes, with four patients having a BRAO and one patient with a BRVO. On imaging the point of occlusion was closely associated with a focus of chorioretinal inflammation [7]. Several theories have been postulated for the association of *B. henselae* infection and retinal vascular occlusion. *B. henselae* has a propensity to invade vascular endothelium and is thought to induce vascular occlusion either through a direct obliterative vasculitis from the organisms themselves or via vascular endothelial damage resulting in thrombogenesis and vaso-occlusion. An intense, focal, inflammatory response may also result in a mechanical obstruction. Optic disc swelling leading to vascular compression has also been implicated [6, 7, 9, 12].

Cases involving CRVO are much more limited. Only two cases of CRVO associated with *B. henselae* infection in adults and one possible case in a child have been reported with 2 of the three cases reporting a history of cat exposure [9, 10, 13]. Both adult patients initially presented with classic ocular signs of *B. henselae* infection and subsequently developed CRVO along with broader, severe, ocular ischemic disease in the absence of treatment. Ghadiali *et* al. reported a patient who initially presented with optic neuritis, peripapillary hemorrhage and macular star formation with initially negative *Bartonella* serologies. Repeat evaluation revealed elevated *B. henselae* IgG titers (1:256) with the subsequent exam showing development of central retinal vein occlusion, concurrent choroidal ischemia and ischemic retinopathy that improved without treatment [9]. Gray and colleagues described a patient who presented with optic disc edema in the setting of illness and cervical and preauricular lymphadenopathy 4 weeks prior. The patient subsequently developed an exudative macular star and reported being previously scratched by a kitten, *B. henselae* IgG titers were elevated (1:128). The patient was non-compliant with antibiotic therapy and developed a combined central retinal artery occlusion and CRVO with neovascular glaucoma [9, 10].

The mainstay of laboratory diagnosis of CSD is serological testing, however local seroprevalence can make interpretation challenging as healthy persons may have low titers of *B. henselae* [6]*.* Seroprevalence rates differ amongst countries, with seropositivity seen in up to 32.38% in Eastern China, 13.7% in Brazil, 5% in New Zealand, and 10.3% in vulnerable populations in the United States and Europe [14–16]. IgG titers greater or equal to 1:256 on serologic testing confirms presence of CSD, and to our knowledge this is the first reported case of *B. henselae* associated CRVO with titers above this threshold, suggesting an active or recent infection [14, 17, 18]. While the presence of IgM antibodies is also useful to detect acute infection, its utility is limited by variable or limited sensitivities [14]. Our case adds to the scarce literature showing that not only is CRVO an exceedingly rare manifestation of ocular bartonellosis, but as in our patient it can be the presenting clinical finding, be associated with acute or recent infection, occur in the absence of cat exposure or broader ocular ischemic disease, and have excellent visual recovery with prompt treatment.

A CRVO in an adult under 40 years-old warrants a thorough workup of inflammatory and infectious etiologies, including a careful history and inquire of risk factors for *B. henselae *[19]. Importantly, while prior exposure to cats and/or prodromal symptoms aids in diagnosis of ocular bartonellosis, they are not required to have the disease. Furthermore, the presence of a CRVO can mask or confound the ability to detect classic signs of ocular bartonellosis such as neuroretinitis due to overlapping features such as optic nerve and macular edema. Therefore, a low threshold for serological testing for *B. henselae* is warranted in this demographic in order to start prompt antibiotic therapy in addition to anti-VEGF agents to maximize visual recovery.

## Data Availability

Data sharing is not applicable to this article as no datasets were generated or analyzed during the current study.

## Abbreviations

*B. henselae*: *Bartonella henselae*
CSD: Cat-scratch disease
anti-VEGF: Anti-vascular endothelial growth factor
BCVA: Best corrected visual acuity
OS: Left eye
CRVO: Central retinal vein occlusion
OD: Right eye
OCT: Optical coherence tomography
BRAO: Branched retinal artery occlusion
BRVO: Branched retinal vein occlusion
CME: Cystoid macular edema
